# Adjunctive therapy with the Tie2 agonist Vasculotide reduces pulmonary permeability in *Streptococcus pneumoniae* infected and mechanically ventilated mice

**DOI:** 10.1038/s41598-022-19560-3

**Published:** 2022-09-15

**Authors:** Aina Lask, Birgitt Gutbier, Olivia Kershaw, Geraldine Nouailles, Achim D. Gruber, Holger C. Müller-Redetzky, Steven Chackowicz, Douglas A. Hamilton, Paul Van Slyke, Martin Witzenrath

**Affiliations:** 1 Department of Infectious Diseases and Respiratory Medicine, Charité—Universitätsmedizin Berlin, corporate member of Freie Universität Berlin and Humboldt-Universität zu Berlin, Charitéplatz 1, 10117 Berlin, Germany; 2 Department of Veterinary Pathology, Freie Universität Berlin, Berlin, Germany; 3 Vasomune Therapeutics, Sunnybrook Research Institute, S-Wing Rm 227, 2075 Bayview Avenue, Toronto, ON M4N3M5 Canada; 4 German Center for Lung Research, (DZL), Gießen, Germany

**Keywords:** Preclinical research, Translational research

## Abstract

Community acquired pneumonia, mainly caused by *Streptococcus pneumoniae* (*S.pn.),* is a common cause of death worldwide. Despite adequate antibiotic therapy, pneumococcal pneumonia can induce pulmonary endothelial hyperpermeability leading to acute lung injury, which often requires mechanical ventilation (MV) causing ventilator-induced lung injury (VILI). Endothelial stabilization is mediated by angiopoietin-1 induced Tie2 activation. PEGylated (polyethylene glycol) Tie2-agonist Vasculotide (VT) mimics Angiopietin-1 effects. Recently, VT has been shown to reduce pulmonary hyperpermeability in murine pneumococcal pneumonia. The aim of this study was to determine whether VT reduces lung damage in *S.pn.* infected and mechanically ventilated mice. Pulmonary hyperpermeability, immune response and bacterial load were quantified in *S.pn.* infected mice treated with Ampicillin + /−VT and undergoing six hours of MV 24 h post infection. Histopathological lung changes, Tie2-expression and -phosphorylation were evaluated. VT did not alter immune response or bacterial burden, but interestingly combination treatment with ampicillin significantly reduced pulmonary hyperpermeability, histological lung damage and edema formation. Tie2-mRNA expression was reduced by *S.pn.* infection and/or MV but not restored by VT. Moreover, Tie2 phosphorylation was not affected by VT. These findings indicate that VT may be a promising adjunctive treatment option for prevention of VILI in severe pneumococcal pneumonia.

## Introduction

Community acquired pneumonia (CAP) is the leading infectious cause of death worldwide^1,2^. Bacterial CAP is most commonly caused by *Streptococcus pneumoniae* (*S. pn.*)^3^. Although antibiotic resistance in CAP is rare, hospitalized patients face a short-term mortality of 10–14%, and up to 35% in severe CAP requiring mechanical ventilation (MV)^4^. Severe pneumonia can cause pulmonary hyperpermeability resulting in pulmonary edema and eventually acute respiratory distress syndrome (ARDS)^5,6^. For lung failure, only supportive therapies are available, and particularly pulmonary barrier disruption lacks therapeutic strategies^7,8^. In addition to antibiotics, MV is key for treating pneumonia-associated ARDS^9^. Yet, lifesaving MV causes further damage to the lungs, known as ventilator-induced lung injury (VILI)^10,11^. Despite the implementation of lung-protective ventilation strategies in clinical practice such as low tidal volume ventilation^12,13^ VILI still occurs particularly in pre-damaged lungs^14,15^.

Angiopoietins 1 and 2 (Ang-1, -2) are ligands for receptor tyrosine kinase Tie2^16,17^. Under inflammatory conditions, Ang-1 improves endothelial integrity and reduces vascular permeability^18,19^ while Ang-2 acts as functional antagonist on Tie2 further aggravating inflammation and destabilizing the endothelial barrier^18,20,21^. Moreover, Tie2 expression is reduced in systemic inflammation further contributing to endothelial destabilization^22^. Ang-2 levels are significantly elevated in patients with pneumonia^23^ and sepsis-associated ARDS^24^ predicting severe or even fatal disease progression^25–27^, while Ang-1 levels are reduced in sepsis^28^ and pneumonia^23^.

Restoring the disturbed balance between phosphorylated and non-phosphorylated Tie2, the synthetic polyethylene glycol (PEG) clustered Tie2 agonist Vasculotide (VT)^29^ has been shown to reduce endothelial permeability in models of abdominal sepsis^30^, hemorrhagic shock^31^, cardiopulmonary bypass^32^, influenza^33^, and pneumococcal pneumonia^34^, even improving survival in influenza and abdominal sepsis^30,33^. In both pneumonia studies—influenza and pneumococcal pneumonia model, respectively—VT did not alter host immune response and pathogen burden^33,34^. As we envision that VT will primarily be developed for use in critical care patients, we investigated VT as therapy in severe murine pneumonia requiring MV. Mice with severe pneumonia were treated with antibiotics and VT, or placebo, and were mechanically ventilated. Lung permeability, immune response, bacterial load, histopathological changes and both Tie2 gene expression and phosphorylation were evaluated.

## Methods

In general, the study complies with local and national guidelines and is in accordance with relevant guidelines and regulations.

### Animals

Female 8–10 weeks old C57BL/6 N mice (Charles River, Sulzfeld, Germany) weighing between 18 and 20 g were used for the experiments. All animal experiments were approved by institutional (“Tierschutzbeauftragte” (animal care taker) and “Tierschutzausschuss” of Charité–Universitätsmedizin Berlin; https://tierschutz.charite.de/tierschutz_an_der_charite/) and governmental (Landesamt für Gesundheit und Soziales Berlin; approval ID A0050/15) authorities and were in accordance with the Federation of European Laboratory Animal Science Associations (FELASA) guidelines and recommendations for the care and use of laboratory animals, which is equivalent to American ARRIVE. Therefore, our animal study meet the reporting requirements laid out in the ARRIVE guidelines. Mice were randomly assigned to the appropriate experimental groups and were kept in closed, individually ventilated cages with filter hoods (type II-L, ZOONLAB), under specific pathogen-free conditions, with free access to food and water, room temperature between 20 and 22 °C, air humidity between 50 and 65% and 12 h light/dark cycle.

### Murine pneumococcal pneumonia and Vasculotide/ampicillin treatment

Mice were anesthetized with an intraperitoneal (i.p.) injection of ketamine (80 mg/kg) and xylazine (25 mg/kg) in a total volume of 60 µl followed by intranasal inoculation of 5 × 10^6^ colony-forming units (CFU) *S. pn.* serotype 3 (NCTC7978) in 20 µl sterile PBS. Control groups received the same volume of PBS.

In a previous study, pulmonary hyperpermeability in experimental pneumococcal pneumonia was reduced by intravenous (i.v.) administration of 500 ng/100 µl VT (Vasomune Therapeutics, Toronto, Canada)^34^. Accordingly, 500 ng/100 µl VT or PBS as control were applied into the lateral tail vein 22 h post infection (p.i.). At this time, a single dose of ampicillin (0.02 mg/g body weight (bw) in 200 µl) was administered i.p. Control groups received the same volume of 0.9% saline.

### Mechanical ventilation

Twenty-four hours p.i., mice were anesthetized with fentanyl (0.08 mg/kg bw), midazolam (8 mg/kg bw) and medetomidine (0.8 mg/kg bw) and ventilated for 6 h via a tracheal cannula (miniVent, Hugo-Sachs Elektronik, March-Hugstetten). A catheter in the left carotid artery, an i.p. catheter and a transurethral catheter were placed as described^35,36^. Ventilation was set to a tidal volume of 12 ml/kg bw, respiratory rate of 120/min, PEEP of 2 cm H_2_O and 50% fraction of inspired oxygen (FiO_2_). During ventilation, mice received continuous infusion (350 µl/h) of full electrolyte solution jonosterile mixed with 0.0075 mmol/ml trometamol and 0.0005 mg/ml fentanyl. Additionally, medetomidin (0.16 mg/kg bw) and midazolam (1.6 mg/kg bw) were administered i.p. to maintain anaesthesia. Oxygen saturation (MouseOX Plus, STARRLife-Scienses, Oakmont, USA), heart rate, blood pressure and tracheal peak pressure (PULMODYN, Hugo-Sachs Elektronik) were monitored throughout MV.

At 4.5 h ventilation time, a second dose of VT or solvent mixed with 1 mg human serum albumin (HSA; Human Serum albumin solution (20%) from CSL Behring (King of Prussia, Pennsylvania, USA) diluted with 0,9% saline) was infused via the carotid catheter. Ten min prior to the end of the experiment, FiO_2_ was increased to 100% and anesthesia was deepened. Five minutes later, 100 µl of heparin at a concentration of 2,500 IU/ml were administered via the carotid catheter.

After 6 h of ventilation mice were exsanguinated. The whole lung was perfused with NaCl 0.9% followed by bronchoalveolar lavage (BAL) of the right lung with proteinase inhibitor. The left lung was removed for flow cytometric analysis.

In non-ventilated control animals, only the six-h ventilation phase was omitted. Anesthesia and preparation were performed 30 h p.i., while HSA with VT or solvent was injected 1.5 h before into one of the lateral tail veins.

### Pulmonary vascular leakage

Pulmonary hyperpermeability was calculated as HSA BALF/Plasma ratio with the corresponding concentrations detected by HSA ELISA (Human Albumin ELISA Kit, Bethyl Laboratories, USA) as described^6^.

### Histological analysis

Separate experiments were performed for histological analysis with 4 mice per experimental group. Mice were treated as described above but did not receive HSA with the second dose of VT. After exsanguination of the mouse the trachea was ligated, lungs and all other organs were removed immediately, fixed in 4% formaldehyde for 24–48 h, embedded in paraffin, cut into 2-μm sections and stained with hematoxylin and eosin as described^37,38^. Lung damage due to pneumonia and VILI was scored by an experienced, board certified veterinary pathologist. Lungs of all animals from the three experimental groups were examined in a blinded manner. Depicted are lungs with changes representative for each group (Fig. 2A). The degrees of VILI or pneumonia severity and of alveolar and perivascular edema formation were assessed semiquantitatively (0 = no damage, 1 = minimal damage/edema, 2 = mild damage/edema, 3 = moderate damage/edema, 4 = severe damage/edema). Three evenly distributed sections per lung were microscopically evaluated to assess edema formation.

### Bacterial loads

Bacterial burden was quantified in bronchoalveolar lavage fluid (BALF) and blood by determining colony forming units (CFU) per ml as described^39^.

### Pulmonary leukocyte differentiation

To assess local immune response, leukocytes in BALF and lung tissue were discriminated by flow cytometry (Canto 2; BD, Heidelberg, Germany) via forward vs. side scatter characteristics and CD11b (M1/70, PE-Cy7, 0.2 mg/ml, BD, Heidelberg), CD11c (N418, Cy5, 1 mg/ml, ATCC, Manassass, Virginia, USA), CD45 (30-F11, FITC, 0.5 mg/ml, BD, Heidelberg), F4/80 (BM8, PE, 0.2 mg/ml, Thermo Fisher Scientific, Waltham, Massachusetts, USA), Ly6G (1A8, PerCP-Cy5.5, 0.2 mg/ml, BD, Heidelberg) and Siglec F (E50-2440, BV421, 0.5 mg/ml, BD, Heidelberg) staining.

### Blood leukocyte differentiation

Blood leukocytes were quantified using a scil Vet abc hematology analyzer (Scil animal care company GmbH, Viernheim, Germany).

### Cytokine multiplex assay

Mouse cytokines/chemokines in blood and BALF supernatant were measured using a Multiplex assay according to the manufacturer´s instructions (ProcartaPlex Multiplex Immunoassay, Thermo Fisher Scientific, USA).

### Tie2 expression

Tie2 expression was analyzed by quantitative reverse-transcriptase polymerase chain reaction (qPCR Mastercycler personal, 7300 rt PCR System, Applied Biosystems, Foster City USA) using RNA extracted from the lavaged right lung. β-2-microtubulin served as housekeeping control. To ensure that an increase in gene expression is accompanied by a positive numerical value, the relative quantification (RQ) values were calculated as follows: RQ = 2^− ΔΔCt. According to this, the arithmetic mean of the control group corresponded to an RQ of 1. An increase in gene expression was accompanied by RQ values > 1, while a reduction in gene expression resulted in RQ values < 1.

### Tie2 phosphorylation

Tie2 phosphorylation was detected by Western Blot analysis of proteins extracted from the lavaged right lung. Snape frozen lung tissues were homogenized in RIPA buffer containing 1 mM NaVO_3_ (Natriummetavanadat), 1 mM NaF (Natriumfluorid) and a proteinase inhibitor (cOmplete, Mini Protease Inhibitor Cocktail, Roche Diagnostics GmbH, Mannheim, Germany) using a gentleMAC dissociator (Miltenyi Biotec, Bergisch Gladbach, Germany). After two centrifugation steps, protein levels in the supernatant were calculated with the DC Protein Assay (BioRad Laboratories, Inc. Hercules, USA) using a 96 well-plate reader. Western Blots were performed with 50 µg protein per band using an 8% gel and semi-dry blotting on a nitrocellulose membrane. The membrane was cut above the 50 kDa band and the two sections were stained over night for actin as loading control (lower membrane section, bands between 50 and 37 kDa; antibody: Actin polyclonal-goat-anti-mouse, Santa Cruz Biotechnology, Dallas, USA) and phospho-Tie2 (upper membrane section, bands between 250 and 75 kDa; antibody: pTie2 (Y992) rabbit-anti-mouse; R&D Systems, Minneapolis, USA) or Tie2 (antibody: Tie2 goat-anti-mouse; R&D Systems, Minneapolis, USA), respectively. Then counterstained for 1 h with the corresponding secondary antibody and developed with an infrared imaging system (LI-COR Odyssey Classic Infrared Imaging System, Biosciences, Lincoln, Nebraska, USA) using a scan intensity of 5 for the wavelengths 700 and 800 nm. To show all bands in an adequate manner only contrast and brightness of the scan imagines were optimize with the associated software Image Studio (LI-COR Biosciences). Densitometry analysis for quantification was performed with the same software. The ratios between Tie2 or pTie2 to actin were calculated.

The Tie2 staining and an afresh actin staining was performed after 30 min incubation with stripping buffer for detaching the antibodies from the membrane.

### Statistical analysis

GraphPad Prism 6 (San Diego, California, USA) was used for the statistical analyses. Results are presented as median and interquartile range (IQR) with the respective minimum and maximum values. VT-treated groups are depicted in light grey in contrast to control groups in dark grey. Comparisons between two groups were performed using the Mann–Whitney-U-Test. Since only directly comparable experimental groups were tested as planned comparisons for the effect of VT therapy, correction for multiple testing was not applied. Effects of MV and *S.pn.* infection on pulmonary permeability, histological damage and immune response^6,34,36^ as well as beneficial effects of ampicillin therapy^40^ have already been described before and were not subject of this study. Corresponding groups only served as methodological controls, therefore no statistical testing was performed between two groups without VT therapy. *p*-values < 0.05 were considered statistically significant.

### Ethics approval and consent to participate

In general, the study complies with local and national guidelines and is in accordance with relevant guidelines and regulations. All animal experiments were approved by institutional (“Tierschutzbeauftragte” (animal care taker) and “Tierschutzausschuss” of Charité–Universitätsmedizin Berlin; https://tierschutz.charite.de/tierschutz_an_der_charite/) and governmental (Landesamt für Gesundheit und Soziales Berlin; approval ID A0050/15) authorities and were in accordance with the Federation of European Laboratory Animal Science Associations (FELASA) guidelines and recommendations for the care and use of laboratory animals, which is equivalent to American ARRIVE. Therefore, our animal study meet the reporting requirements laid out in the ARRIVE guidelines.

## Results

### Combination therapy with Vasculotide and ampicillin reduced vascular hyperpermeability in mechanically ventilated mice with pneumonia

**Figure 1:**
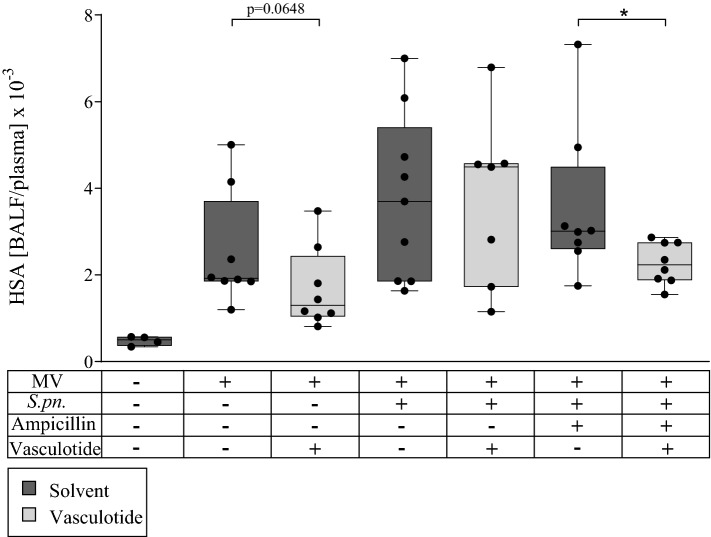
Combination treatment of VT and ampicillin reduced pulmonary permeability 30 h p.i. Vasculotide (VT) (500 ng/100 µl intravenously (i.v.)) or phosphate buffered saline (PBS), and ampicillin (0.4 mg/kg bw intraperitoneally (i.p.)) or 0.9% saline were administered 22 h after *Streptococcus pneumonia (S. pn.)* infection (control: PBS + hyaluronidase). The 6-h ventilation phase began 24 h post infection (p.i). A second dose of VT together with 1 mg human serum albumin (HSA) was administered 1.5 h prior to termination of the experiment. Cell-free bronchoalveolar lavage fluid (BALF)/plasma ratio for HSA was calculated as a measure of permeability. Values are listed as median + IQR with minimum and maximum values, individual values are shown as dots (control n = 4, all other groups n = 7–9; *p < 0.05, Mann–Whitney-U-Test).

Vascular hyperpermeability was quantified by calculating HSA BALF/plasma ratio 30 h p.i. (Fig. 1). Permeability values of non-ventilated, non-infected mice without any therapy served as reference value (hereinafter referred to as the control group). Ventilation of non-infected animals led to an increase in permeability with VT-therapy tending to a reduced permeability. MV in mice with pneumonia appeared to result in higher vascular permeability indicating more pronounced barrier disruption than MV in uninfected mice. VT monotherapy did not significantly reduce permeability in mechanically ventilated mice with pneumonia, while combination therapy with ampicillin resulted in a significant reduction of lung permeability compared to ampicillin monotherapy.

### Combination therapy with Vasculotide and ampicillin reduced histological lung damage in mechanically ventilated mice with pneumonia

**Figure 2:**
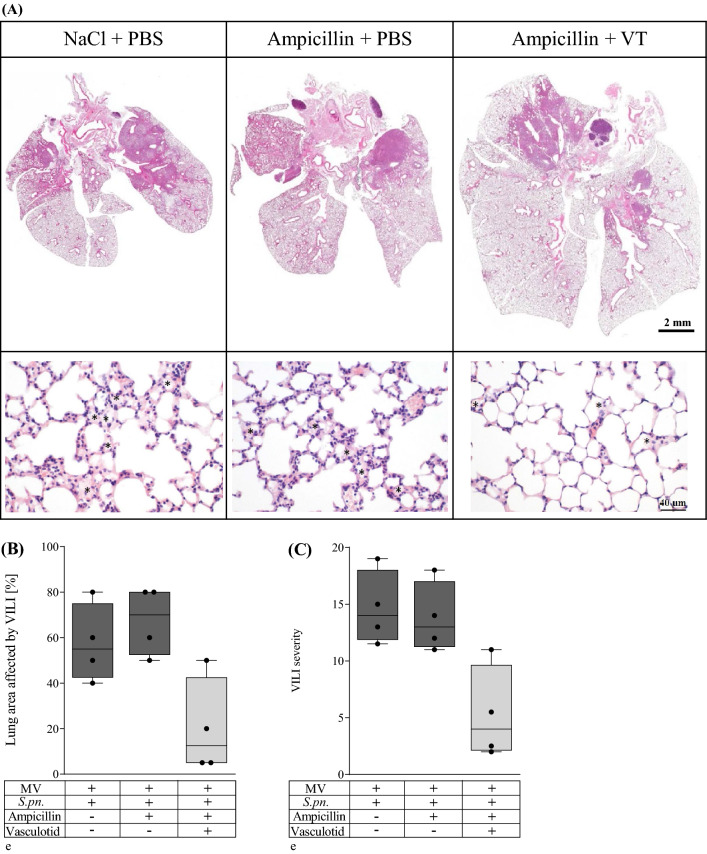

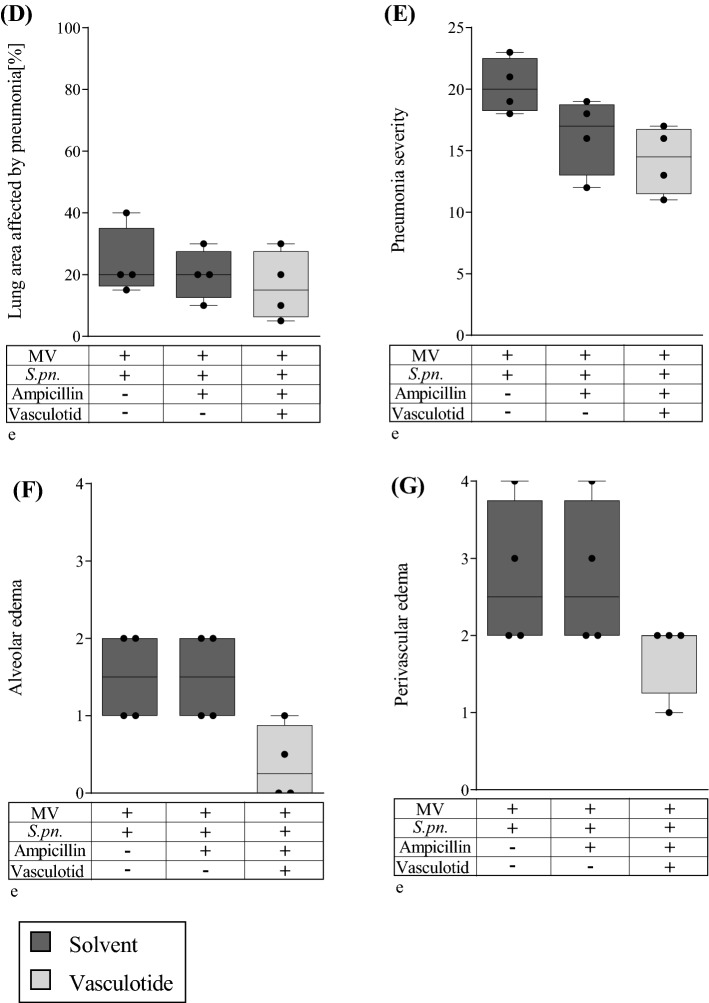
Combination treatment of VT and ampicillin improved histological lung damage. VT (500 ng/100 µl i.v.) or PBS, and ampicillin (0.4 mg/kg bw i.p.) or 0.9% saline were administered 22 h after *S. pn.* infection (control: PBS + hyaluronidase). The 6-h ventilation phase began 24 h p.i. A second dose of VT was administered 1.5 h prior to termination of the experiment. Histological features of pneumonia and VILI were assessed semiquantitatively by scoring (0 = no damage, 1 = minimal damage, 2 = mild damage, 3 = moderate damage, 4 = severe damage). (**A**) Combined treatment with VT was associated with higher lung volumes, fewer consolidated areas and diminished edema formation (asterisks in bottom panels). Representative images of centrally placed cross sections of whole lungs are shown. (**B**, **D**) Percentage of lung area affected by VILI (**B**) or pneumonia (**D**). (**C**, **E**) Severity of VILI (**C**) and pneumonia (**E**) in affected lung areas represented by an overall histological score (VILI 10 features, max. score 40; pneumonia 5 features, max. score 20). (**F**, **G**) Alveolar edema formation in VILI-affected lung areas (**F**) and perivascular edema formation in pneumonia-affected lung areas (**G**). Values are listed as median + IQR with minimum/maximum values, individual values are shown as dots (n = 4).

Histological analysis was carried out on infected, mechanically ventilated animals without treatment, with ampicillin monotherapy and with VT/ampicillin combination therapy. Lungs of VT and ampicillin treated animals had higher lung volumes and fewer consolidated areas than animals treated with ampicillin alone or receiving no therapy, as observed in macroscopic as well as microscopic assessments (Fig. 2A).

Furthermore, morphologic changes were evaluated using histological scores (Fig. 2B–G). Because pneumonic infiltrates potentially mask ventilation-induced pathologic changes, VILI was analyzed only in areas devoid of pneumonic infiltrates. VT in combination with ampicillin distinctively reduced the area of lung tissue affected by VILI (Fig. 2B) and the severity of VILI in these areas (Fig. 2C). In contrast, therapy with ampicillin alone showed no effect compared to untreated animals. Neither ampicillin monotherapy nor combination with VT had a beneficial effect on the percentage of lung tissue area affected by pneumonia (Fig. 2D) or severity in these lung areas (Fig. 2E). However, treatment with ampicillin and VT considerably reduced alveolar edema formation in VILI-affected lungs (Fig. 2A,F) and perivascular edema formation in pneumonia-affected lungs (Fig. 2G) in comparison to antibiotic therapy-only or untreated mice.

### Vasculotide did not affect the pulmonary or systemic cellular immune response in mechanically ventilated mice with pneumonia

**Figure 3:**
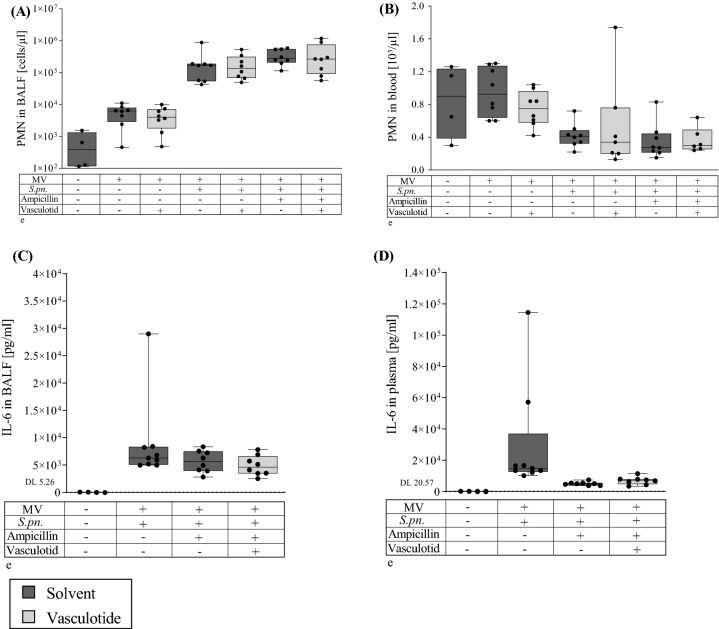
Pulmonary and systemic immune response was not altered by VT therapy 30 h p.i. Vasculotide (VT) (500 ng/100 µl i.v.) or PBS, and ampicillin (0.4 mg/kg bw i.p.) or 0.9% saline were administered 22 h after Streptococcus pneumonia (*S. pn*.) infection (control: PBS + hyaluronidase). The 6-h ventilation phase began 24 h post infection (p.i). A second dose of VT together with 1 mg HSA was administered 1.5 h prior to termination of the experiment. Immune cells in the alveolar space were isolated from BALF, stained with antibodies binding leukocyte surface markers and differentiated by flow cytometry. Blood leukocytes were analyzed with a hemocytometer. Cytokines in BALF and plasma were measured by Multiplex Cytokine assay. (**A**) Neutrophils (PMN) per µl BALF, (**B**) Neutrophilis per µl blood, (**C**) IL-6 in BALF (pg/ml) and (**D**) IL-6 levels in plasma (pg/ml). Values are listed as median + IQR with minimum/maximum values, individual values are shown as dots (control n = 4, all other groups n = 7–8, Mann–Whitney-U-Test).

To study the pulmonary and systemic immune responses, leukocytes in BALF and blood were quantified and differentiated. MV-induced sterile inflammation was associated with an increase in neutrophils in BALF (Fig. 3A), which was more pronounced after prior *S. pn.* infection. Recruitment of neutrophils remained unaffected by ampicillin, VT or combination of both. In contrast to BALF, the number of neutrophilic granulocytes in the blood was reduced in infected and ventilated animals (Fig. 3B). Neither monotherapy with either VT or ampicillin, nor combination therapy improved leukocytopenia.

### Vasculotide did not modulate production of pro- or anti-inflammatory cytokines and chemokines in mechanically ventilated mice with pneumonia

For further assessment of local and systemic inflammation, cytokines (GM-CSF, IL-1β, IL-6, IL-12p40 and IL-10) as well as chemokines (KC and MIP-2) were quantified in BALF and plasma by multiplex cytokine assay.

*S. pn.* infection and MV increased local cytokine and chemokine production measured in BALF (Fig. 3C for IL-6, Supplemental Figure S1 A–F for other cytokines/chemokines). VT in combination with ampicillin had no modulatory effect on local cytokine or chemokine concentrations. Higher concentrations of the anti-inflammatory cytokine IL-10 were observed in BALF of some animals treated with ampicillin and VT, yet results are too scattered to conclude a tendency. In systemic circulation, cytokine and chemokine concentrations were reduced by antibiotics, but this reduction was not enhanced by additional VT therapy (Fig. 3D for IL-6, Supplemental Figure S1 G–L for other cytokines/chemokines).

### Vasculotide had no effect on local or systemic bacterial burden in mechanically ventilated mice with pneumonia

**Figure 4:**
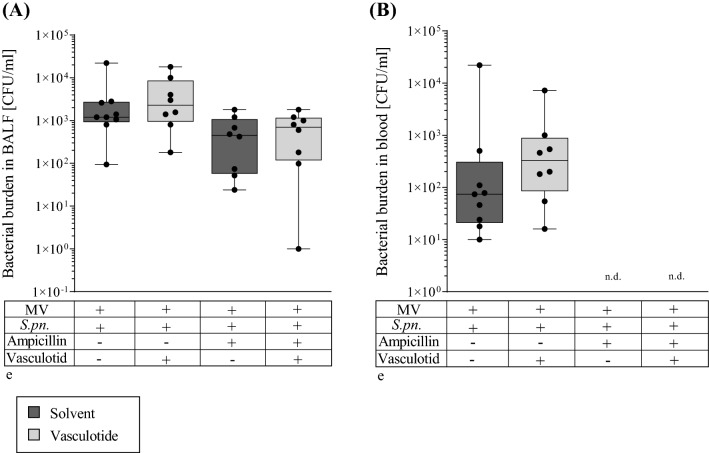
Pulmonary and systemic bacterial burden was not altered by VT therapy 30 h p.i. Vasculotide (VT) (500 ng/100 µl i.v.) or PBS, and ampicillin (0.4 mg/kg bw i.p.) or 0.9% saline were administered 22 h after Streptococcus pneumonia (*S. pn*.) infection (control: PBS + hyaluronidase). The 6-h ventilation phase began 24 h post infection (p.i). A second dose of VT together with 1 mg HSA was administered 1.5 h prior to termination of the experiment. Bacterial burden in BALF and blood was quantified by plating both samples in increasing dilutions on sheep blood agar, counting CFU and thus determining concentration per µl. (**A**) Bacterial burden in BALF and in blood (**B**). Values are listed as median + IQR with minimum/maximum values, individual values are shown as dots (all groups n = 7–8, Mann–Whitney-U-Test).

To determine the effect of Vasculotide on bacterial burden in the alveolar space and on bacteremia, we counted CFUs in BALF and blood, respectively (Fig. 4A,B). Ampicillin-therapy distinctly reduced bacterial burden in BALF and completely eradicated bacteria from systemic circulation. Bacterial burden was not affected by additional or sole VT-therapy.

### Pulmonary Tie2-receptor expression was reduced by pneumonia and MV

Tie2-receptor expression in the lung was evaluated using qPCR (supplemental figure S2). MV alone reduced Tie2-expression compared to the control group. *S. pn.* infection with or without MV further lowered Tie2 levels. Among the groups of infected animals, no differences were found between ventilated and non-ventilated animals. VT in combination with ampicillin or the ampicillin monotherapy did not alter Tie2-expression in infected animals.

### Pulmonary Tie2 receptor phosphorylation was not increased by Vasculotide

**Figure 5:**
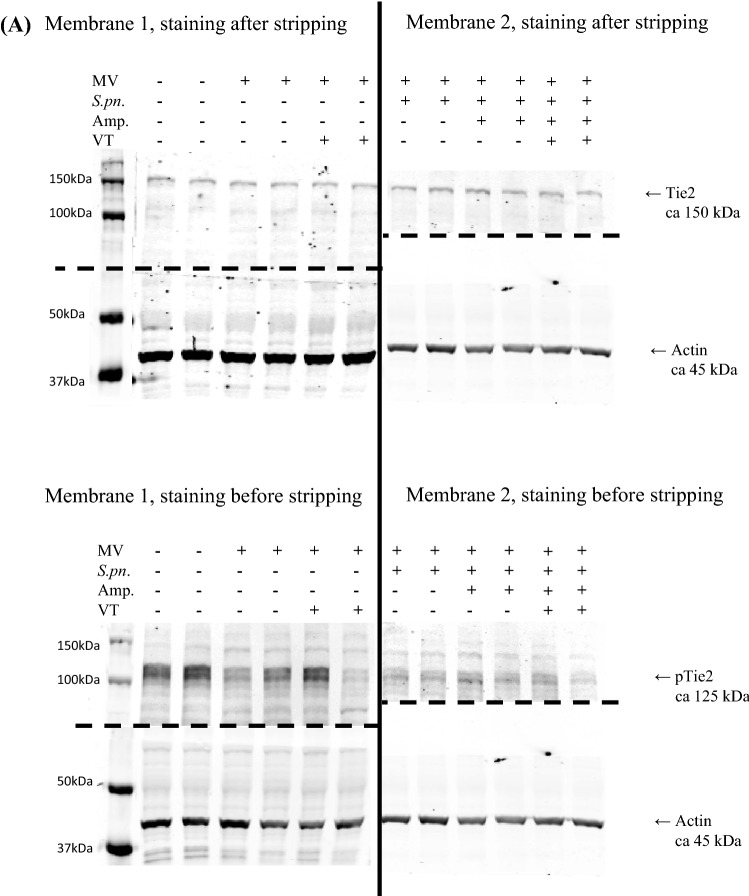

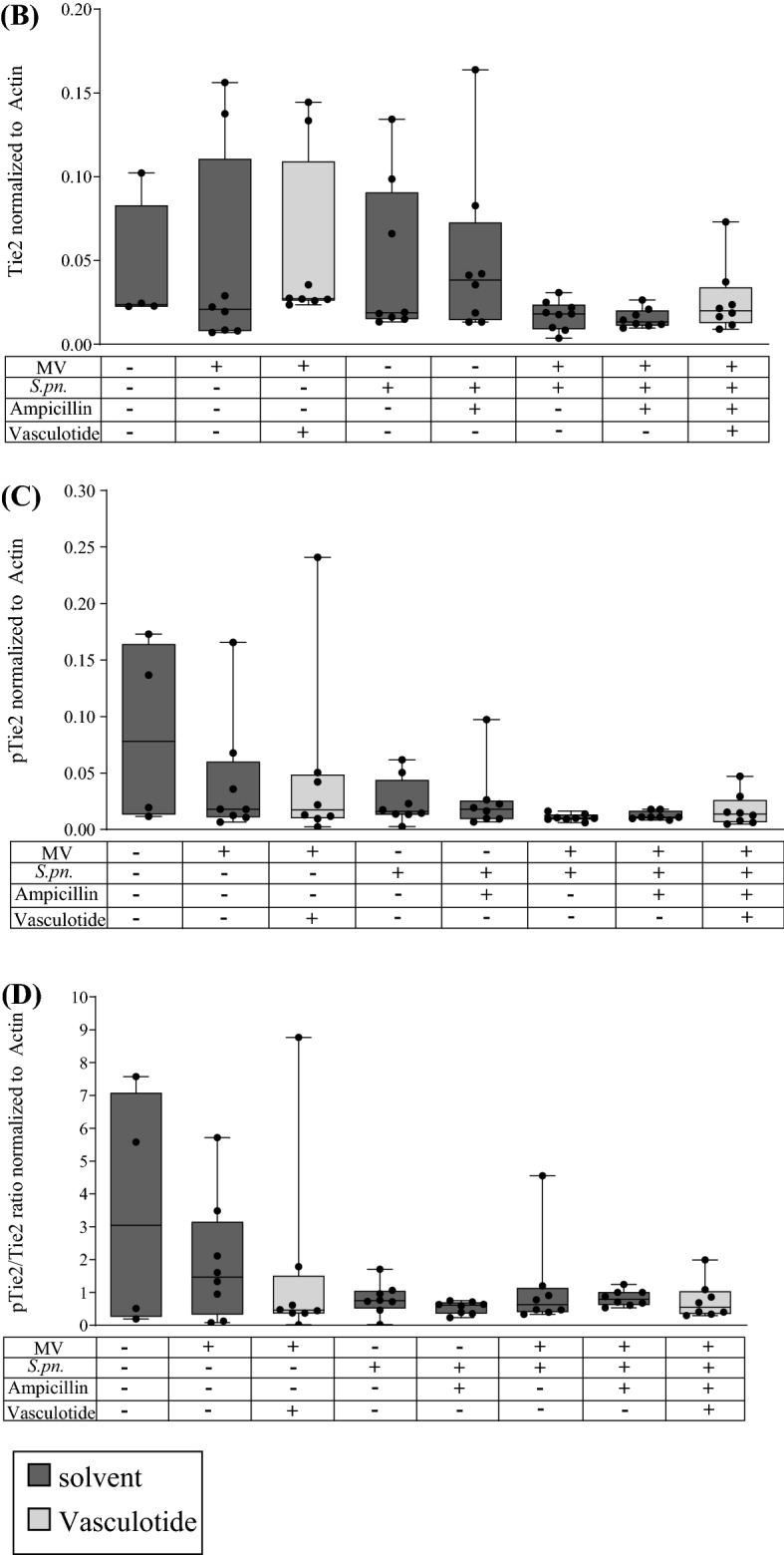
Protein expression and phosphorylation of Tie2 receptor were not influence by VT treatment 30 h p.i. Mice were intranasally infected with *S. pneumoniae* (*S. pn;* 5 × 10^6^ colony-forming units per mouse) or sham-infected as control with phosphate buffered saline ((PBS) + hyaluronidase). Twenty-two h after infection, Vasculotide (VT) (500 ng/100 µl i.v.) or PBS, and ampicillin (0.4 mg/kg bw i.p.) or 0.9% saline were administered and 24 h post infection (p.i.) mice were ventilated for 6 h. A second dose of VT together with 1 mg human serum albumin (HSA) was administered 1.5 h prior to finishing the experiment. (**A**) To assess Tie2-phosphorylation, proteins were isolated from lung homogenates and Western blot was performed with antibodies for phospho-Tie2 (pTie2), Tie2 (staining after incubation with stripping buffer for detaching the pTie2 antibody from the membranes) and actin as loading control, and developed by infrared imagine. Representative blots for all groups are shown: Membrane 1 (left images) for the non-infected groups, membrane two (right images) for the infected groups. The black line separates the two membranes, dotted lines show the areas where the membranes were cut in two sections before staining. After staining both sections were developed together by infrared imagine. For quantification, the ratio between Tie2 and actin densitometry (**B**) and pTie2 and actin densitometry (**C**) was calculated, respectively. The quotient of pTie2 to Tie2 (**D**) was used to estimate the phosphorylation status of the receptor. Values are listed as median ± IQR with minimum/maximum values, individual values are shown as dots (control n = 4, all other groups n = 8–9, Mann–Whitney-U-Test).

Analyzing protein isolated from lung homogenates, VT treatment had no significant effects on Tie2 receptor phosphorylation (Fig. 5A–D). Compared to non-ventilated, non-infected animals, the qualitative and quantitative evidence of phospho-Tie2 decreased due to infection and/or MV. Further, we failed to observe a Tie2-activation after VT-administration.

## Discussion

In severe murine pneumococcal pneumonia without additional ventilation, we previously observed reduction of pulmonary hyperpermeability by VT^34^ as well as by early antibiotic therapy^40^. The present study shows that in *S. pn.* infection combined with MV neither one of the two monotherapies is effective, whereas combination therapy with ampicillin and VT significantly reduces pulmonary permeability and histological lung damage without relevantly suppressing the immune system or affecting the bacterial burden. In our model, pneumonia and ventilation synergistically enhanced inflammation and permeability^36^. We here again observed that MV induces VILI in mice with pneumonia, which adds to pneumonia-evoked lung damage. For C57BL/6 N mice, a tidal volume of 12 ml/kg is most “protective”, as lower tidal volumes would require higher breathing frequencies resulting in even more lung damage^36^. In this clinically relevant scenario, only the combination of both antibiotic therapy with ampicillin and adjunctive therapy with VT was efficient in reducing permeability, further supporting the notion that mechanically ventilated patients with severe pneumonia may benefit from adjunctive barrier-stabilizing therapies^41^.

We assume that, in case of additional ventilation, the inflammatory lung damage is so severe that monotherapy with VT or ampicillin can no longer be effective. In our study the different mechanisms of damage seem to reinforce each other, an observation which is also supported by results of Müller-Redetzky et al*.* demonstrating that lung damage induced by combination of pneumonia and MV leads to a significantly higher inflammation (measured by cytokines and chemokines) as well as permeability than mere addition of these values for infection and ventilation alone^36^. Treatment with ampicillin primarily leads to reduction of systemic inflammation and bacterial dissemination, resulting in reduced systemic cytokine concentrations and lower bacterial burden in the blood and thus stabilizing the pulmonary barrier^40^. However, ampicillin, has no effect on sterile inflammation induced by MV. VT seams to act via direct stabilization of the endothelium without effect on inflammatory mediators and thus could be used in both sterile as well as bacterial inflammation to improve endothelial integrity. Since both agents convey their protective effects through different mechanisms it could be reasonable that a combination might be able to restore efficacy in a situation where respective monotherapies fail.

The formation of pulmonary edema in pneumococcal pneumonia is primarily promoted by two detrimental factors: the disruption of blood-air barrier by bacteria and their virulence factors^6^, and the increased endothelial permeability due to host immune response involving myeloid and non-myeloid cells, cytokines and chemokines released after activation of pathogen recognition receptors (PRRs) resulting in the recruitment of neutrophils and macrophages^5,42^.

In this study, VT did not affect leukocyte recruitment, cytokine secretion or bacterial burden. Similar results have previously been published for pneumococcal and influenza virus pneumonia^33,34^. However, other studies have demonstrated a reduction in neutrophil influx and cytokine concentrations^30,43–45^. These studies have used different routes of application and prophylactic rather than therapeutic treatments, suggesting that potential immunomodulatory effects of VT may depend on the timing of its initial administration. It is further tempting to speculate that the treatment duration in our study was too short to alter the cellular immune response.

Previous work by David and colleagues has shown a reduction of Tie2 expression in endotoxin-induced acute lung injury^22^, which we also observed in MV, and even more pronounced in pneumonia and pneumonia-associated VILI. VT did not increase Tie2 expression in the previous^22^ or the present study, suggesting that permeability reduction is not mediated by Tie2 induction but rather by increased Tie2 activation. Since VT was generated from a synthetic peptide specifically selected for its binding affinity to Tie2^46^, this conclusion seems justified and has already been supported by different other studies^22,30,33^. However, we were unable to detect such increased activation in vivo in this study, and a study by Wu et al*.*^47^ concluded that VT utilizes Tie2-independent pathways^47^. Nonetheless, the lack of detection of Tie2 phosphorylation might also be due to methodological difficulties. Phosphorylation could have been impaired or lost to some degree during storage at − 80 °C or during protein isolation. As pneumococcal infection strongly decimates Tie2 expression, Western Blot might be a method with insufficient sensitivity to detect differences. Moreover, the time point chosen for our analysis might not be ideal, however, maximum phosphorylation of Tie2 had been observed 20 to 30 min after VT-application in vitro^29^, while David et al. found phosphorylation lasting for 72 to 96 h in vivo^22^, suggesting that 8 h after first and 1.5 h after the last application should be a well-chosen time point. Thus, further research is needed to conclusively determine both Tie2-dependent and possible Tie2-independent VT effects at the cellular level.

## Conclusion

In summary, VT reduced VILI in murine pneumococcal pneumonia without affecting pulmonary and systemic immune response or bacterial elimination. Particularly the formation of alveolar edema was diminished in VT-treated mice. Thus, VT may be a promising adjunctive treatment to prevent lung injury in mechanically ventilated patients with severe pneumonia.

## Supplementary Information

Supplementary Figures.

## Data Availability

Data are available on request.

## Abbreviations

ARDS: Acute respiratory distress syndrome
Ang-1: Angiopoietin-1
Ang-2: Angiopoietin-2
BAL: Bronchoalveolar lavage
BALF: Bronchoalveolar lavage fluid
bw: Body weight
CAP: Community-acquired pneumonia
CFU: Colony-forming units
FiO_2_: Fraction of inspired oxygen
h: Hour
HSA: Human serum albumin
IL: Interleucine
i.p.: Intraperitoneal
i.v.: Intravenous
IQR: Interquartile range
MV: Mechanical ventilation
NaVO_3_: Natriummetavanadat
NaF: Natriumfluorid
PBS: Phosphate-buffered saline
PEEP: Positive end-expiratory pressure
PEG: Polyethylene glycol
*p.i.*: Post infection
RQ: Relative quantification
*S.pn*.: *Streptococcus pneumonia*
VT: Vasculotide
VILI: Ventilator-induced lung injury
