# Supplementary material for: Adjunctive therapy with the Tie2 agonist Vasculotide reduces pulmonary permeability in Streptococcus pneumoniae infected and mechanically ventilated mice
Source: Sci Rep. 2022 Sep 15;12:15531. doi: 10.1038/s41598-022-19560-3 (PMC9478100; doi:10.1038/s41598-022-19560-3)
Supplement: Supplementary file 1 — Supplementary Figures. [file 41598_2022_19560_MOESM1_ESM.docx]

**Adjunctive therapy with the Tie2 agonist Vasculotide reduces pulmonary permeability in *Streptococcus pneumoniae* infected and mechanically ventilated mice**

Aina Lask^*^, Birgitt Gutbier^*^, Olivia Kershaw, Geraldine Nouailles, Achim D. Gruber, Holger C. Müller-Redetzky, Steven Chackowicz, Douglas A. Hamilton, Paul Van Slyke, Martin Witzenrath

*These authors contributed equally

**Supplementary Figures**

**Supplemental Figure S1: Pulmonary and systemic cytokines and chemokines concentrations were not affected by VT treatment 30 h p.i.**

Mice were intranasally infected with *S. pneumoniae* (*S. pn;* 5x10^6^ colony-forming units per mouse) or sham-infected as control with phosphate buffered saline ((PBS) + hyaluronidase). Twenty-two h after infection, Vasculotide (VT) (500 ng/100 µl intravenously (i.v.)) or PBS, and ampicillin (0.4 mg/kg bw intraperitoneally (i.p.)) or 0.9 % saline were administered and 24 h post infection (p.i.) mice were ventilated for 6 h. A second dose of VT together with 1 mg human serum albumin (HSA) was administered 1.5 h prior to finishing the experiment. The cytokines and chemokines levels in bronchoalveolar lavage fluid (BALF; A-F) and plasma (G-L) were measured by Multiplex Cytokine assay. GM-CSF (A+G), IL-1β (B+H), Il-12p40 (C+I) and IL-10 (D+J) as well as chemokines KC (E+K) and MIP-2 (F+L). Values are listed as median + IQR with minimum/maximum values, individual values are shown as dots (n.d.= non detectible; control n=4, all other groups n=7-8, Mann-Whitney-U-Test).

**Supplemental Figure S2: Expression of Tie2 receptor were not influence by VT treatment 30 h** **p.i**

Mice were intranasally infected with *S. pneumoniae* (*S. pn;* 5x10^6^ colony-forming units per mouse) or sham-infected as control with phosphate buffered saline ((PBS) + hyaluronidase). Twenty-two h after infection, Vasculotide (VT) (500 ng/100 µl i.v.) or PBS, and ampicillin (0.4 mg/kg bw i.p.) or 0.9 % saline were administered and 24 h post infection (p.i.) mice were ventilated for 6 h. A second dose of VT together with 1 mg human serum albumin (HSA) was administered 1.5 h prior to finishing the experiment. To assess gene expression of the Tie2 receptor, qPCR was performed with cDNA samples obtained from RNA isolated from lung homogenates.. Values are listed as median ± IQR with minimum/maximum values, individual values are shown as dots (control n=4, all other groups n=8-9, Mann-Whitney-U-Test).
